# Sugar-based bicyclic monomers for aliphatic polyesters: a comparative appraisal of acetalized alditols and isosorbide

**DOI:** 10.1080/15685551.2016.1231038

**Published:** 2016-10-16

**Authors:** Elena Zakharova, Antxon Martínez de Ilarduya, Salvador León, Sebastián Muñoz-Guerra

**Affiliations:** ^a^ Departament d’Enginyeria Química, Universitat Politècnica de Catalunya, ETSEIB, Barcelona, Spain; ^b^ Departamento de Ingeniería Química, Universidad Politécnica de Madrid, ETSIIM, Madrid, Spain

**Keywords:** Biobased polyesters, sustainable polyesters, aliphatic polyesters, sugar based polyesters, acetalized alditols, isosorbide, D-glucose, D-mannose, D-sorbitol, D-glucitol

## Abstract

Three series of polyalkanoates (adipates, suberates and sebacates) were synthesized using as monomers three sugar-based bicyclic diols derived from D-glucose (Glux-diol and isosorbide) and D-mannose (Manx-diol). Polycondensations were conducted in the melt applying similar reaction conditions for all cases. The aim was to compare the three bicyclic diols regarding their suitability to render aliphatic polyesters with enhanced thermal and mechanical properties. The ensuing polyesters had molecular weights (*M*_w_) in the 25,000–50,000 g mol^−1^ range with highest values being attained for Glux-diol. All the polyesters started to decompose above 300 °C and most of them did not display perceivable crystallinity. On the contrary, they had glass transition temperatures much higher than usually found in homologous polyesters made of alkanediols, and showed a stress–strain behavior consistent with their *T*_g_ values. Glux-diol was particularly effective in increasing the *T*_g_ and to render therefore polyesters with high elastic modulus and considerable mechanical strength.

## 1. Introduction

Plastic production today is currently over 260 Mt and it is still based by far on fossil fuel resources.[1,2] The replacement of oil-based monomers by those others derived from renewable resources (carbohydrates, lignin, vegetal oils) is today one of the most active areas in polymer research.[3–5] A recent study predicts that the worldwide capacity of bio-based plastics will increase from about 0.4 Mt in 2007 to near 3.5 Mt in 2020.[6]

Polyesters are definitely among the most promising families of renewable polymers because the building-blocks needed for their synthesis, both diols and diacids, are relatively easily accessible.[7] Although aromatic polyesters (PET, PBT, etc...) are predominant at the industrial scale, aliphatic polyesters are rapidly increasing in importance due to their unique ability to combine a satisfactory performance with a good biocompatibility and significant biodegradability. Although ROP is the polymerization procedure usually preferred for producing some relevant aliphatic polyesters (poly(L-lactic acid), polycaprolactone, etc...), most polyesters are usually obtained by melt polycondensation of alkanediols and either *α*,*ω*-alkanedioic acids or their methyl esters.[8] Carbohydrates are particularly suitable as raw materials for producing diols and diacids for renewable polymers, and in fact, a good number of them are today available at medium or large scale, thanks to the recent advances achieved in fermentation technology.

Carbohydrate-based diols and diacids with a bicyclic structure are particularly interesting building-blocks for polyesters synthesis because they are unique in increasing chain stiffness and hence conferring them high glass transition temperatures.[9,10] Isohexides are a family of alditols dianhydrides with a fused bis-tetrahydrofurane structure that are prepared by dehydration of aldohexitols. The isohexide coming from D-glucose (dianhydro-1,4:3,6-D-glucitol) is commonly known as isosorbide, abbreviated Is, and it is currently produced on industrial scale. This compound is widely used in sustainable polymer chemistry, and in particular for the modification of aliphatic and aromatic polyesters by replacing the alkanediol in more or less extent.[11–13] Literature is plenty of references to polycondensation polymers and copolymers made from Is, and several Is containing copolyesters have achieved commercialization.[14–16] In these last years, another family of based-carbohydrate bicyclic monomers has emerged. This comprises among others, alditols and aldaric acids methylene diacetals with a structure made of two fused 1,3-dioxane rings. The bicyclic diacetalized hexitols derived from D-mannose and D-glucose (2,4:3,5-di-*O*-methylene-D-mannitol and -D-glucitol), abbreviated as Manx-diol and Glux-diol, have been extensively explored as monomers for the synthesis of aromatic and aliphatic copolyesters.[10,17] The results reported on these monomers have clearly shown that they may be efficiently used for melt polycondensation and that they are able to enhance the thermal and mechanical properties of polyesters while providing them with a significant biodegradability.[18,19]

**Scheme 1.**
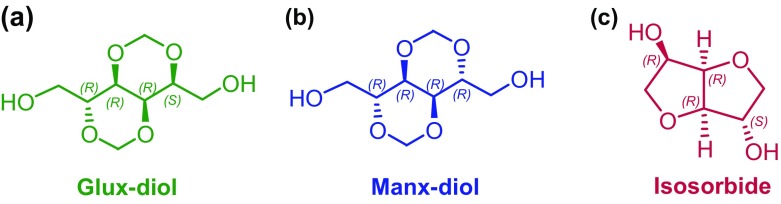
Molecular structures of Glux-diol (a) Manx-diol (b) and Is (c).

Isosorbide and bicyclic diacetalized alditols differ in that the free hydroxyl groups are secondary in the former and primary in the later. Furthermore Is and Glux-diol are non-symmetrical molecules whereas Manx-diol contains a twofold axis (Scheme 1). Such structural differences are largely expected to be reflected in the polymers resulting from their use as polycondensation monomers. In fact, a couple of papers have been published in which the two types of sugar-based bicyclic compounds are compared as comonomers regarding their suitability for the synthesis and properties of PET and PBT copolyesters.[20,21] In the present work we wish to report a study comparing Glux-diol, Manx-diol and Is for the synthesis of aliphatic homopolyesters by polycondensation in the melt. Three series of polyalkanoates made from dicarboxylic acids with 6, 8 and 10 carbon atoms have been chosen for this study. The value of this work relies on the high sustainable character of the sugar-based homopolyesters that are prepared as well as on the enhancing effect that the bicyclic structure exerts on their glass-transition temperature.

## 2. Experimental part

### 2.1. Materials

The reagents dimethyl adipate (DMAdi) (≥99%), dimethyl suberate (DMSub) (99%), diethyl sebacate (98%) (DESeb), 1,4:3,6-dianhydro-D-glucitol (Is) (98%) and the catalyst dibutyl tin oxide (DBTO) (98%), were purchased from Sigma-Aldrich. Solvents used for purification, synthesis and characterization were all of either technical or high-purity grade and they were purchased from Panreac and used as received without further purification. Irganox 1010 and Irgafos 126 antioxidants were a generous gift from BASF. The cyclic diols 2,4:3,5-di-*O*-methylene-D-glucitol (Glux-diol) and 2,4:3,5-di-*O*-methylene-D-mannitol (Manx-diol) were prepared according to the procedures described by us.[21,22]

### 2.2. General methods

^1^H and ^13^C NMR spectra were recorded on a Bruker AMX-300 spectrometer at 25.0 °C operating at 300.1 and 75.5 MHz, respectively. About 10 or 50 mg of polyester for ^1^H and ^13^C NMR, respectively, were dissolved in 1 mL of deuterated chloroform and spectra were internally referenced to tetramethylsilane (TMS). Sixty-four scans for ^1^H and 1000–10,000 scans for ^13^C with 32 and 64-K data points with relaxation delays of 1 and 2 s, respectively, were registered. Viscosities of polyesters were measured in dichloroacetic acid at 25.00 ± 0.01 °C, using an Ubbelohde capillary viscometer at concentrations ranging from 0.5 to 1 g dL^−1^. Gel permeation chromatograms were acquired at 35.0 °C on a Waters equipment provided with a refraction-index detector. The samples were chromatographed with 0.05 M sodium trifluoroacetate-hexafluoroisopropanol mixture (NaTFA-HFIP) using a PL HFIP gel column (300 × 7.5 mm) with a flow rate of 0.5 mL min^−1^. Chromatograms were calibrated against poly(methyl methacrylate) (PMMA) monodisperse standards. The thermal behavior of polyesters was examined by DSC using a Perkin Elmer DSC8000 apparatus from 3 to 5 mg samples at heating/cooling rates of 10 °C min^−1^ and under a nitrogen flow of 20 mL min^−1^. Indium and zinc were used as standards for temperature and enthalpy calibrations. The glass-transition temperatures were determined by the tangent method at a heating rate of 20 °C min^−1^ from rapidly melt-quenched polymer samples. Thermogravimetric analyses were performed in a Mettler Toledo TGA/DSC 1 thermobalance under a nitrogen flow of 20 mL min^−1^ at a heating rate of 10 °C min^−1^ within a temperature range of 30 to 600 °C. Sample weights of about 10–15 mg were used in these experiments. Films for mechanical properties with a thickness of ~200 μm were prepared by either casting or hot-pressing. Samples for testing were cut into strips with a width of 3 mm while the distance between testing marks was 10 mm. The Young’s modulus, tensile strength, and elongation at break were measured at room temperature applying a stretching rate of 30 mm min^−1^. A Zwick 2.5/TN1S testing machine coupled with a compressor Dalbe DR 150 was used for these essays. X-ray diffraction patterns from powdered samples coming from synthesis were recorded on the PANalytical X’Pert PRO MPD *θ/θ* diffractometer using the CuK*α* radiation of wavelength 0.1542 nm.

### 2.3. Polyester synthesis

The three sets of sugar-based polyesters examined in this work, which amounts a total of nine different polymers, with indication of the monomers they come from, as well as the abbreviations used to designate them, are listed in Table 1. They all were synthesized by melt polycondensation of mixtures composed of the corresponding alkanedioic dialkyl ester and bicyclic diol using similar reaction conditions for all cases. It should be noted that 10%-mol excess of diol respect to diester was routinely used to compensate possible evaporation losses. Reactions were carried out in a three-necked, round bottom flask equipped with a mechanical stirrer, a nitrogen inlet and a vacuum distillation outlet. The reactants were stirred to get a homogeneous mixture, to which DBTO (0.4–0.6 mol% respect to the total of monomers) was added as catalyst.

**Table 1.** Aliphatic polyesters studied in this work.

Alkylenediester	Sugar-based bicyclic diol		
	Glux-diol	Manx-diol	Is
		
Dimethyl adipate	PGluxAdi	PManxAdi	PIsAdi

Dimethyl suberate	PGluxSub	PManxSub	PIsSub

Diethyl sebacate	PGluxSeb	PManxSeb	PIsSeb


The antioxidants Irganox 1010 (0.2% w/w) and Irgafos 126 (0.6% w/w) were then added to minimize possible degradation of the thermally sensitive sugar-based monomers. As usual, polymerization was made to proceed through two steps. Transesterification reactions taking place in the first step were conducted at temperatures increasing from 160 up to 200 °C for a period of time oscillating between 3 and 6 h depending on the case. A nitrogen flow was applied at this step to keep the pressure around one bar in order to minimize volatilization of diols and avoid oxidation of the sugar-based compounds. Polycondensation reactions occurring in the second step were performed at 180–220 °C for 3–8 h. High vacuum (0.03–0.06 mbar) was applied at this stage with the object of favoring the removal of byproducts and attaining high conversions. When the reaction was terminated, the reaction mixture was cooled to room temperature under a nitrogen flow to prevent degradation. The solidified mass was dissolved in chloroform, and the polymer precipitated with methanol, collected by filtration and dried under vacuum. The chemical constitution and purity of the prepared polyesters were ascertained by NMR.

## 3. Results and discussion

### 3.1. Synthesis and chemical structure

The bicyclic acetalized alditols 2,4:3,5-di-*O*-methylene-D-glucitol (Glux-diol) and 2,4:3,5-di-*O*-methylene-D-mannitol (Manx-diol) were prepared from commercially available 1,5-D-gluconolactone respectively, in satisfactory yields and with the purity required for polycondensation. The methods used for these syntheses have been previously reported by us [21,22]; a scheme of the involved reactions is provided in the ESI document.

**Scheme 2.**
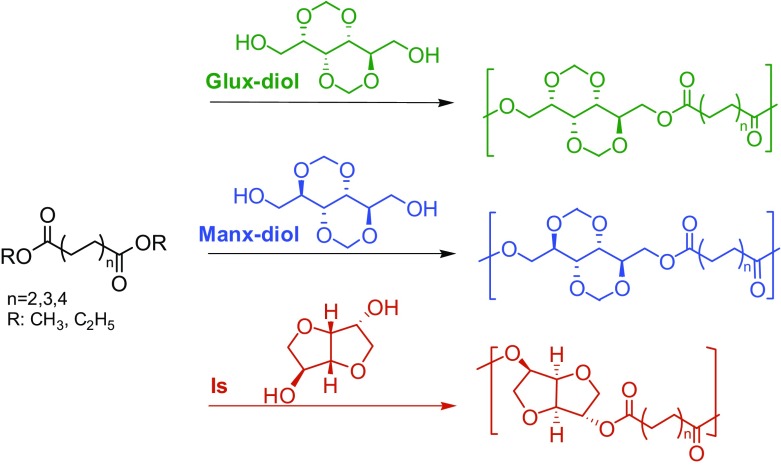
Polycondensation reactions leading to aliphatic polyesters.

Polycondensation reactions were performed according to Scheme 2. As it is usually done, the process consisted of two steps, *i.e.*, generation of hydroxyl capped oligoesters by transesterification followed by polycondensation under vacuum to remove the excess of diol and low molecular weight byproducts. Reaction conditions were optimized for each individual system and for the two steps with the purpose of reaching high molecular weights without apparent decomposition of the sugar-based compounds. A detailed account of temperature and time values applied at each case is given in Table 2. Polycondensation proceeded invariably with a continuous increase in viscosity of the reaction mixture, which usually became so high as to impede stirring.

**Table 2.** Molecular weights and reaction conditions selected for the preparation of aliphatic polyesters.

Polyester	Molecular weights				Reaction conditions	
	[*η*]^a^(dL g^−1^)	*M*_n_^b^(g mol^−1^)	*M*_w_^b^(g mol^−1^)	*Ð*^b^	Transesterification (N_2_)	Polycondensation (0.03–0.06 mm)
PGluxAdi	0.57	20,100	41,300	2.1	180 °C; 4 h	180–210 °C; 3 h
PGluxSub	0.71	20,900	46,500	2.2	180 °C; 4 h	180–220 °C; 4 h
PGluxSeb	0.69	16,700	34,400	2.1	170 °C; 6 h	180 °C; 4 h
PManxAdi	0.50	10,400	29,000	2.8	160 °C; 3 h	180–210 °C; 8 h
PManxSub	0.69	16,000	41,600	2.6	160 °C; 3 h	180–215 °C; 4 h
PManxSeb	0.70	13,300	36,200	2.7	160 °C; 3 h	180–215 °C; 6 h
PIsAdi	0.48	10,100	27,400	2.7	180–200 °C; 5 h	220 °C; 8 h
PIsSub	0.68	16,900	38,400	2.3	180 °C; 3 h	200–215 °C; 6 h
PIsSeb	0.68	13,000	34,000	2.6	180 to190 °C; 3 h	210–220 °C; 6 h

^a^ Intrinsic viscosity measured in dichloroacetic acid at 25 °C.

^b^ Determined by GPC in HFIP against PMMA standards.

**Figure 1.**
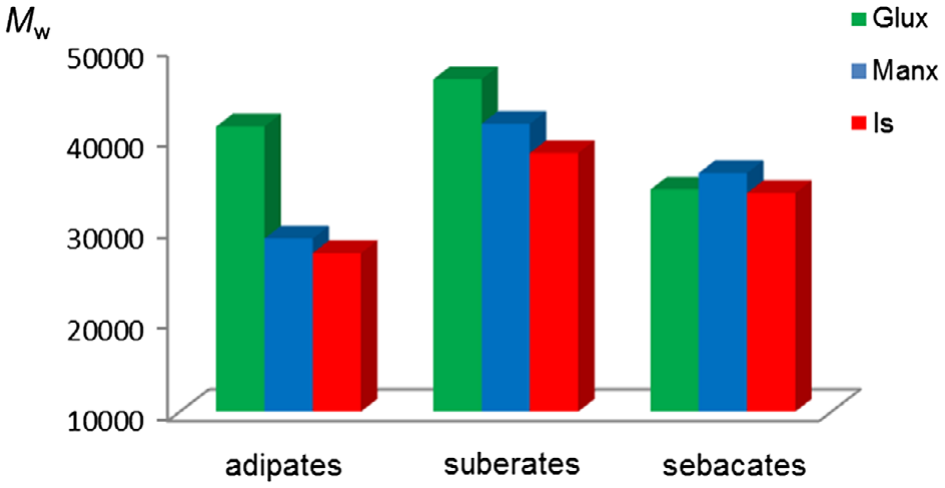
Compared weight-average molecular weight of aliphatic polyesters made from Glux-diol, Manx-diol and Is.

The chemical constitution and purity of all polyesters was ascertained by both ^1^H and ^13^C NMR spectroscopy. The recorded spectra together with their respective signal assignments are accessible in the ESI file (Figures SI-1–SI-5). The GPC analysis revealed that they were obtained with weight-average molecular weights within the 50,000–25,000 range and molar mass dispersities oscillating between 2 and 3. Intrinsic viscosities varied from 0.5 and 0.7 dL g^−1^ in good agreement with the trend observed for molecular weights (Table 2). Molecular weights deserve particular attention due to their relevance to the properties of the polyesters, and also because their values are in some way an indication of the reactivity of monomers. It is widely known that the difficulty in obtaining high enough molecular weights is a general drawback in the synthesis of polyesters which becomes critical when sugar-based monomers are concerned. For a more vivid comparative illustration, the weight-average molecular weight values obtained for the three series of the bicyclic sugar-based polyesters are represented in Figure 1 as a bar graphic. Although no clear correlation beween constitution and molecular weights can be appreciated with a general validity, some valuable conclusions may be drawn when molecular weights are compared regarding the bicyclic diol used for building the polyesters. Whereas differences between Glux and Manx containing polyesters are not entirely systematic, maximum *M*_w_ values were attained for adipates and suberates when Glux-diol was the chosen monomer. Furthermore, polyesters made from Is invariably show the lowest molecular weights in the all three groups. These results are in agreement with those obtained when polyterephthalate copolyesters prepared from similar bicyclic sugar-based monomers were compared.[10]

The fact that the two free hydroxyl groups of Is are secondary rationalizes the lower reactivity displayed by this monomer compared to both Glux-diol and Manx-diol, each one bearing two hydroxymethyl groups. On the contrary, the differences in molecular weights observed between Glux and Manx containing polyesters are certainly striking. The two hydroxymethyl groups in the Manx-diol are spatially identical due to the *C*_2_ symmetry of the Manx configuration whereas in the Glux structure such groups are allocated in different spatial positions (*exo* and *endo*). Accordingly, a greater reactivity for Manx-diol, and in consequence a higher molecular weight for the Manx-containing polyester, should be expected. However, the opposite result is found for both polyadipates and polysuberates whereas differences observed for polysebacates are not significant. These results reveal that, in contrast to what is reported for isosorbide,[16] the *exo*-*endo* influence on reactivity must be practically inoperating in Glux-diol. This is in agreement with the fact that a similar number of *exo* and *endo* hydroxyl end groups are detected in the NMR spectra of Glux containing polyesters whereas an excess of *endo* hydroxyl groups are found in the polymers made from Is (Figure SI-6 and SI-7).

### 3.2. Thermal properties and crystallinity

**Figure 2.**
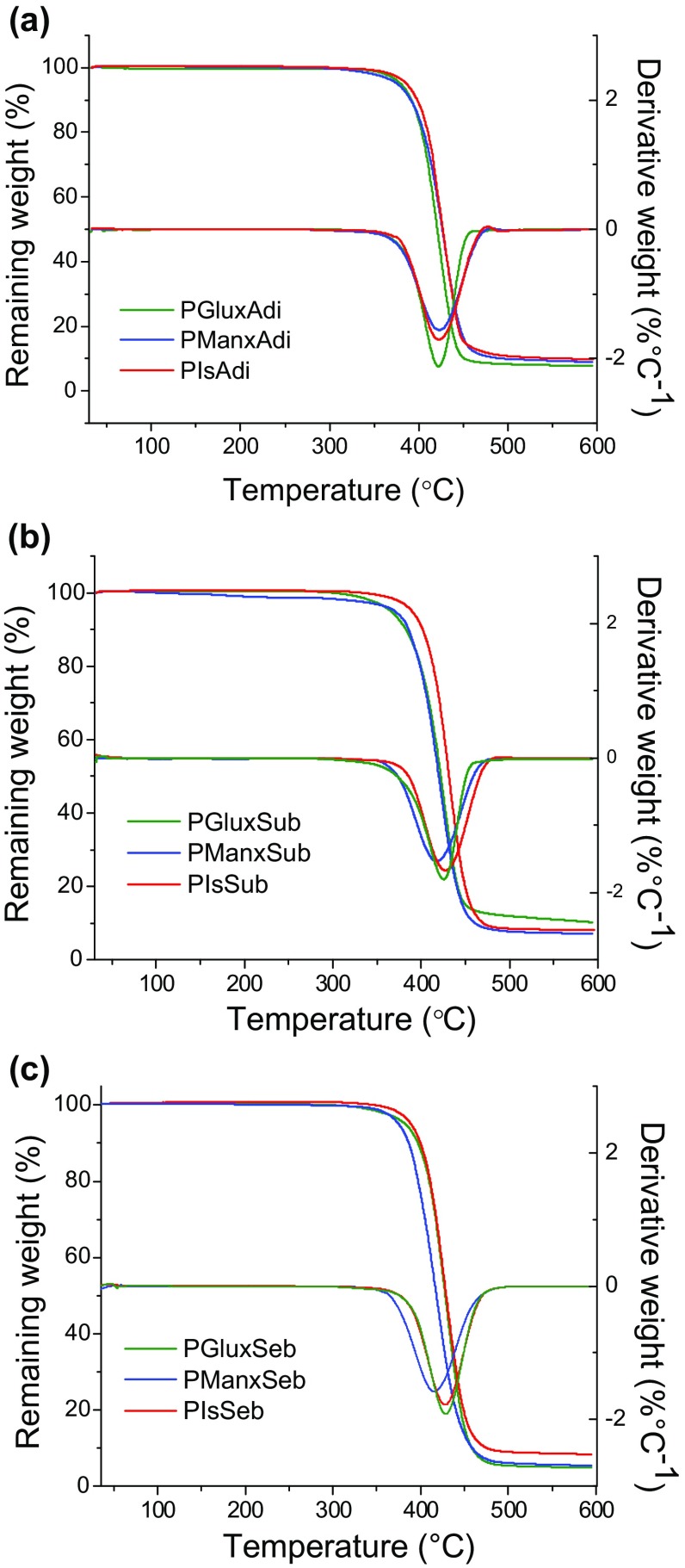
Compared TGA traces of aliphatic homopolyesters made from dimethyl adipate (a) dimethyl suberate (b) diethyl sebacate (c) and its derivative curves.

The thermal behavior of the polyesters was evaluated by TGA and DSC, and the main thermal parameters recorded from these essays are collected in Table 3. The thermal stability was measured in the of 30–600 °C range under a nitrogen flow, and the registered TGA traces are compared in Figure 2. A rapid survey of these data leads to conclude that the aliphatic polyesters containing bicyclic units derived from alditols are stable to temperatures well above ~350 °C and decompose in one step leaving less than ~10% of residue after being heated above ~450 °C. The relevant consequence of such pattern of behavior is that the resistance to heat of these polyesters is high enough as to allow their comfortable processing by conventional thermal methods. A closer comparison of data listed in Table 3 indicates that decomposition takes place at a maximum degradation rate temperature (^max^ *T*_d_), located within the 420–430 °C range with differences among polyesters being less than ~2%. On the other hand, the differences observed for the onset temperature of decomposition, (°*T*_d_), measured for a loss of 5% of the initial weight, are also small although in this case polymers made from Is and Max-diol display respectively the highest and lowest values in every polyalkanoate group. It can be also stated that the thermal stability tends to increase slightly with the length of the alkanoate moiety for whichever sugar-based diol is used in the synthesis of the polyester.

**Table 3.** Thermal properties of sugar-based aliphatic polyesters.

Polyester	TGA^a^			DSC^b^		
	*°T*_d_ (*°*C)	^max^*T*_d_ (*°*C)	RW (%)	*T*_g_ (*°*C)	*T*_m_ (*°*C)	∆*H*_m_ (J g^−1^)
PGluxAdi	380	422	8	72	–	–
PManxAdi	368	422	6	29	–	–
PIsAdi	386	423	10	20	–	–
PGluxSub	366	424	10	57	133	21.2
PManxSub	370	420	7	26	–	–
PIsSub	389	429	8	18	–	–
PGluxSeb	382	429	5	45	117	30.3
PManxSeb	375	420	5	22	–	–
PIsSeb	389	429	9	5	52	20.9

^a^ Onset decomposition temperature corresponding to 5% of weight loss (^°^ *T*_d_), temperature of maximum degradation rate (^max^ *T*_d_), and % of weight remaining after heating at 600 °C (RW).

^b^ Glass-transition temperature (*T*_g_) taken as the inflection point of the heating DSC traces of melt-quenched samples recorded at 20 °C min^−1^. Melting temperatures (*T*_m_) and melting enthalpy (*ΔH*_m_) measured at a heating rate of 10 °C min^−1^ from pristine powdered samples.

**Figure 3.**
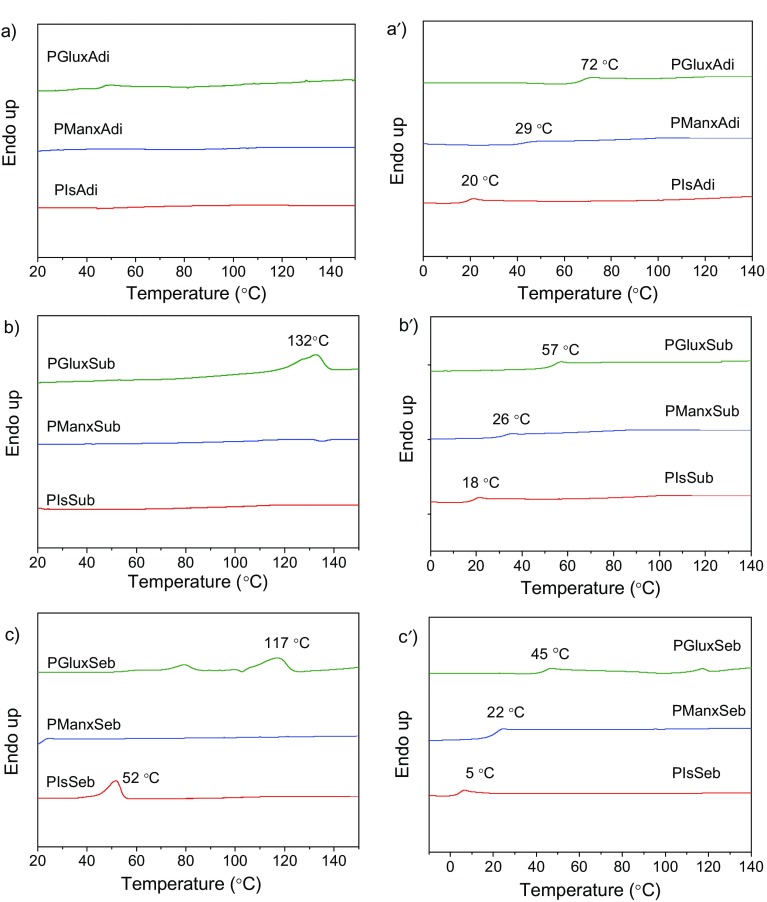
DSC traces of bio-based aliphatic homopolyesters. First heating traces from pristine samples: (a) polyadipates, (b) polysuberates, (c) polysebacates. Heating traces from samples quenched from the melt for *T*_g_ observation: (a’) polyadipates, (b’) polysuberates, (c’) polysebacates.

Comparison of melting and glass transition temperatures of the polyesters has been made on the basis of data collected by DSC. The DSC traces obtained at heating from samples coming directly from synthesis are depicted in Figure 3(a–c) for the three respective sets of polyalkanoates. As a general rule, the presence of sugar-based bicyclic units in the polyester chain entailed a dramatic decrease of crystallinity so that only PGluxSub, PGluxSeb, and PIsSeb displayed DSC containing endothermal peaks characteristic of melting. Conversely, plain traces typical of amorphous material were recorded from the three polyadipates as well as from the three Manx containing polyalkanoates. The greater ability of polyesters to crystallize as the length of the diacid moiety increases is highly predictable and may be explained by the higher spatial flexibility that is provided to the polymer chain. On the other side, the absence of crystallinity in polyesters containing Manx is not easily understandable since the Manx structure is the only one out that is able to render a polymer chain with stereoregular configuration. Nevertheless, what it is most noteworthy is that the Glux structure appears to be the more favorable one to develop crystallinity in aliphatic polyesters. Such effect has been previously observed in aromatic copolyesters containing the same sugar-based bicyclic units.[20,23] Compared to PIsSeb, both PGluxSub and PGluxSeb have much higher melting temperatures and enthalpies. The greater ability of Glux containing polyesters to crystallize compared to those containing either Is or Manx should be related to the greater capacity of the Glux bicyclic structure to become accommodated in an ordered array of chains. However this effect is not easy to explain and its rationalization will require additional efforts.

**Figure 4.**
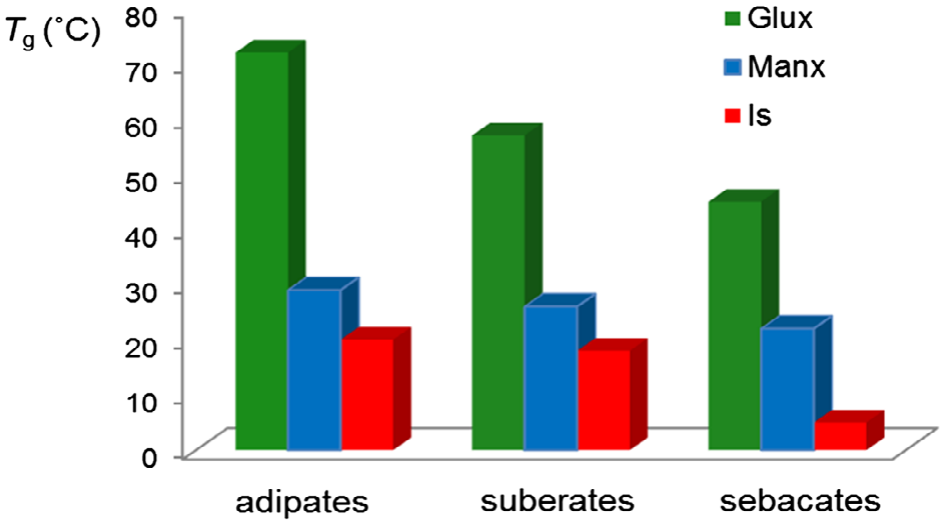
Compared glass transition temperatures of aliphatic homopolyesters made from Glux-diol, Manx-diol and Is.

The glass-transition temperature (*T*_g_) is probably the property most greatly affected by the incorporation of cyclic units in an aliphatic polyester chain. Poly(alkylene alkanoate)s in general suffer from having very low *T*_g_ due to the very high flexibility inherent to the aliphatic polyester chain and they display in consequence poor mechanical properties.[24–26] Both the diacetalized Glux and Manx units as well as Is are highly rigid bicyclic structures that introduce a considerable stiffness in the polyester chain. The *T*_g_ values of the sugar-based bicyclic polyalkanoates examined in this work are listed in Table 3 and represented in the bar graphic of Figure 4 for a more straightforward comparison. As expected, all these polyalkanoates display *T*_g_ values much higher than those of their homologous made of alkanediols, which are frequently below 0 °C.[27,28] A more detailed comparison of the data presented in Figure 4 leads to the following conclusions: (a) The *T*_g_ of polyalkanoates made of a given diol, decreases almost linearly with the increase in length of the alkanoate unit. (b) The polyesters containing bicyclic diacetalized units show *T*_g_ significantly higher than those containing Is. (c) The Glux units are particularly effective in increasing the *T*_g_ of aliphatic polyesters with differences arriving to be larger than 50 °C between PGluxAdi and PIsAdi.

The exceptional effectiveness of the Glux structure to restrict chain mobility, and in consequence to rise the *T*_g_ of the polymer, was brought into evidence for the first time in several studies of aromatic polyesters made recently by us [19,20]; in fact it was then reported that the polyterephthalate made of Glux-diol had a *T*_g_ of 154 °C, which is about 120 and 75 °C higher than those of PBT and PET, respectively. The reason for such strong effect could be attributed to the puckered molecular conformation adopted by the Glux bicyclic configuration compared to the more planar arrangement found in Manx. The bulkiness of Glux should be therefore a factor to be added to cyclic rigidity at explaining the reduction in free volume that apparently occurs in polyesters containing this structure.

**Figure 5.**
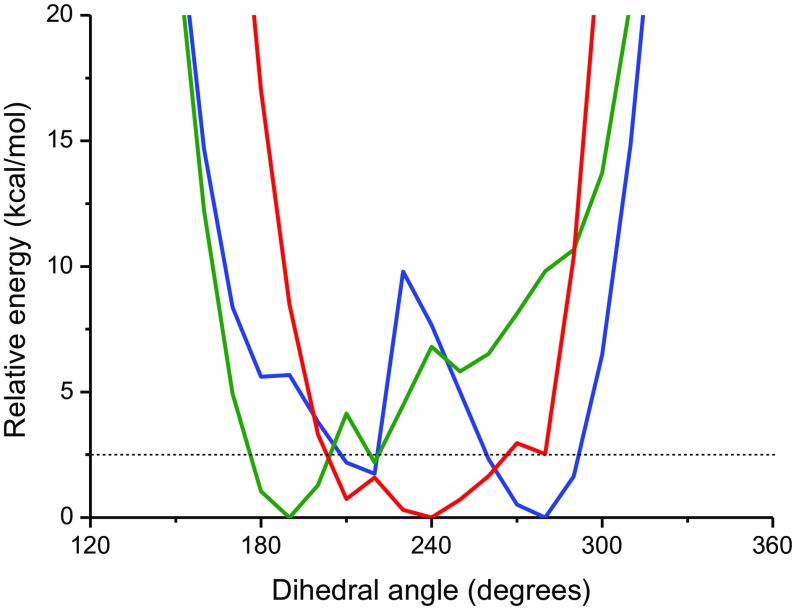
Energy profile obtained for the torsion respect to the central C–C bond for the three diol monomers under study: Glux-diol (green), Manx-diol (blue) and Is (red).

The conformational preferences for Glux-diol, Manx-diol, and Is compounds have been evaluated by density functional theory (DFT) calculations. The molecular geometry of these molecules has been optimized, and the minimum character of such geometries has been verified by a frequency analysis. In addition, the torsional energy profile associated with the central bond of each molecule has been obtained, by fixing the corresponding torsion in intervals of 10º and allowing the rest of coordinates to relax. All the DFT calculations have been performed at the B3LYP/6-31G(d,p) level of theory with the GAMESS program.[29] Figure 5 displays the torsional energy profiles for the rotation around the C–C central bond in the three units. It can be seen that for the three monomers, complete rotation is precluded by the presence of the two fused rings. More specifically, the profiles for the Glux and Manx monomers are narrower than for Is (about 170–250º for Glux and 240–300º for Manx, as compared to about 190–290º for Is), suggesting that the associated rotation will be more restricted for the former two monomers. A larger flexibility could be therefore expected for the polymers based on the Is monomer.

**Figure 6.**
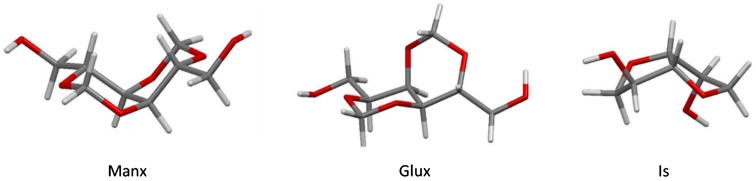
DFT-calculated minimum energy geometries for the three diol monomers under study.

Regarding Glux and Manx, their profiles display a similar width. However, the presence of two energy minima in the Manx profile could account for the comparatively greater flexibility observed for the polyesters containing this structure. Moreover, comparison of the minimum energy conformations reveals geometrical differences for the Glux and Manx units. The most favorable geometry for Manx (Figure 6) is relatively planar with the two 1,3-dioxane ring units in a chair conformation and the two hydroxymethyl groups in equatorial positions. On the other hand, the optimal conformation for Glux corresponds to a puckered geometry with only one of the rings in a chair conformation while the other adopts a twisted boat conformation in order to accommodate its hydroxymethyl group in a non-axial position. Such differences could also contribute to the greater effect of Glux on the reduction of flexibility on its corresponding polymers.

**Figure 7.**
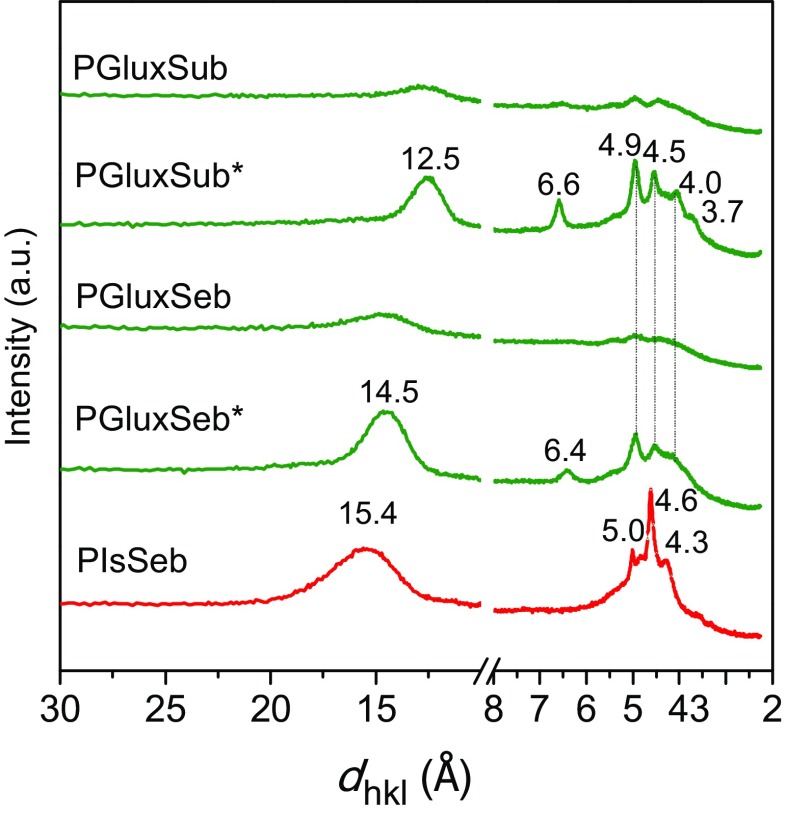
Powder WAXS profiles of PGluxSub, PGluxSeb and PIsSeb homopolyesters coming directly from synthesis.*Profile obtained of PGluxSub annealed at 120 °C for 1 h and PGluxSeb annealed at 105 °C for 1 h.

To support DSC data, the polyesters showing crystallinity were examined by powder X-ray diffraction. The scattering profiles recorded for PGluxSub, PGluxSeb and PIsSeb are depicted in Figure 7 with indication of the Bragg spacings associated to the most prominent observed peaks. Only PIsSeb was able to render a crystalline diffraction pattern from the pristine sample. Sharp strong reflections at 5.0, 4.6, and 4.3 Å were present at the profile of this polyester together with another broad one centered on ~15 Å, which very probably arises from the axial repeat of the structure. In the other two cases, samples had to be subjected to annealing in order to obtain well-defined discrete scattering (Figure SI-8). The profiles recorded from PGluxSub and PGluxSeb annealed samples displayed a similar profile in the wider angle region with peaks at 6.5, 4.9, 4.5, 4.0, and 3.7 Å. The fact that the same diffraction pattern is observed over the 5–3 Å interval may be easily understandable because the reflections appearing therein are usually interpreted as arising from the side-by-side chain packing of the structure, which is expected to be essentially determined by the Glux bicyclic units. On the contrary, the spacing differences observed in the smaller angle region (14.5 and 12.5 for PGluxSeb and PGluxSub, respectively) are in agreement with the different repeat that would result for the packing of suberic and sebacic segments along the axial repeat of the structure. According to results, it seems that it is the packing of the sugar moiety that exclusively determines the geometry of the crystal structure in aliphatic polyesters containing Glux units.

### 3.3. Stress–strain behavior

**Figure 8.**
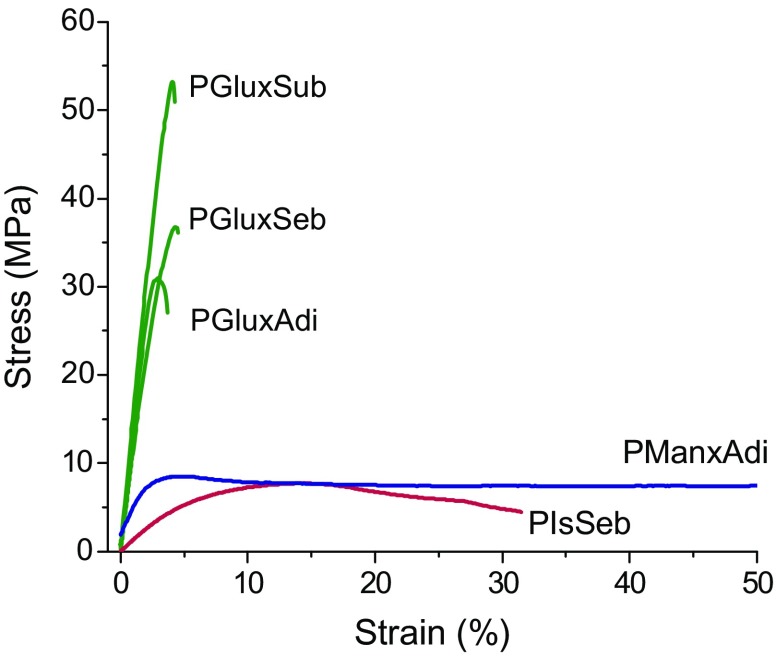
Stress–strain curves of aliphatic polyesters containing sugar-based bicyclic units.

The evaluation of the mechanical properties of the sugar-based bicyclic polyesters was performed by tensile essays of films that were prepared by either hot pressing or casting (Figure 8). Unfortunately, not all the polyesters were evaluated because films suitable for testing could not be always obtained. All the Glux containing polyesters in addition to PManxAdi and PIsSeb could be tested whereas too brittle films unable for measuring under stretching were produced by all others. Given the strong influence that crystallinity is known to exert on mechanical behavior, the films were examined by DSC before being subjected to mechanical testing. DSC data and mechanical parameters obtained in these essays are gathered in Table 4.

**Table 4.** Mechanical properties of aliphatic polyesters.

Polyester	DSC^a^		Stress–strain essays		
	*T*_m_ (°C)	∆*H*_m_ (J g^−1^)	*E* (MPa)	*σ*_max_ (MPa)	*ε* (%)
PGluxAdi	–	–	1522 ± 70	30 ± 3	3 ± 1
PGluxSub	133	19.5	945 ± 10	49 ± 5	5 ± 1
PGluxSeb	115	3.6	1160 ± 20	32 ± 8	4 ± 2
PManxAdi	–	–	243 ± 20	9 ± 3	230 ± 20
PIsSeb	53	19.1	110 ± 10	8 ± 2	34 ± 7

^a^ Melting temperatures (*T*_m_) and enthalpies (*∆H*_m_) of films measured by DSC at heating of 10 °C·min^−1^.

In general it can be stated that polyesters made from Glux-diol display much higher modulus and tensile strength than the others made from either Manx-diol or Is which are in turn much less extensible. This behavior is a clear consequence of their comparatively higher *T*_g_. In fact, Young’s moduli of Glux containing polyalkanoates are within 1.1–1.5 GPa range, which are amazingly high values for aliphatic polyesters. Tensile strengths ranged between 30 and 50 MPa and elongations to break were below 5%, which are usual values found for this type of polymers. On the other hand, the amorphous PManxAdi displayed a much lower modulus and tensile strength than its homologous PGluxAdi but it can be stretched up to more than 200% without breaking. Such great different behavior is doubtlessly due to the lower *T*_g_ displayed by the Manx containing polyadipate. Although only a polyester containing Is could be measured, PIsSeb, comparison of data with its Glux containing homologous, PGluxSeb, revealed mechanical behavior differences consistent with their *T*_g_ values. The polysebacate containing Is results to be a much softer and weaker polymer than that containing Glux although it was able to be stretched in a much longer extent.

## 4. Conclusions

Polymerization results as well as thermal and mechanical properties of the ensuing polyesters were systematically compared for three series of polyalkanoates made from two diacetalized alditols (Glux-diol and Manx-diol) and from a doubly anhydridized glucitol (Is). The diacetalized alditols (Glux-diol and Manx-diol) were clearly superior to Is in producing higher molecular weight polyesters whichever alkanoate was concerned but no consistent differences between the two diacetals were apparent along the three polyester series. All polyesters displayed good thermal stability regardless the type of sugar-based unit involved with no significant differences among them. The repressing effect of the bicyclic alditol on crystallinity became obvious for the three diol monomers but it was less noticeable for Glux-diol. On the contrary, they all showed an enhancing effect on *T*_*g*_ whose intensity follows the order Glux ≫ Manx > Is in the three polyalkanoate series. As a consequence the mechanical behavior of the polyesters changed from strong and hard in those made of Glux-diol to soft and weak in those made of either Manx-diol or Is. The results provided by this study bring into evidence the intrinsic superiority of Glux-diol and Manx-diol on Is for polycondensation with dialkyl dialkanoates as well as for enhancing the thermal and mechanical behavior of the ensuing aliphatic polyesters. The commercial inaccessibility of the diacetalized diols is however a severe handicap in short term for both scientific and technical development of these monomers.

## Disclosure statement

No potential conflict of interest was reported by the authors.

## Funding

This work was supported by MINECO of Spain and from AGAUR [grant number MAT2012-38044-C03-03, 2009SGR1469].

## Supplemental data

Supplemental data for this article can be accessed at http://dx.doi.org/10.1080/15685551.2016.1231038

## Supplementary Material

TDMP_1231038_Supplemental_Material.pdf
