# Supplementary material for: Sugar-based bicyclic monomers for aliphatic polyesters: a comparative appraisal of acetalized alditols and isosorbide
Source: Des Monomers Polym. 2016 Oct 16;20(1):157–66. doi: 10.1080/15685551.2016.1231038 (PMC5814662; doi:10.1080/15685551.2016.1231038)
Supplement: TDMP_1231038_Supplemental_Material.pdf [file TDMP_A_1231038_SM3127.pdf]

Electronic Supplementary Information

Sugar-based bicyclic monomers for aliphatic polyesters: A comparative appraisal of acetalized alditols and isosorbide

Elena Zakharova,[1] Antxon Martínez de Ilarduya,[1] Salvador León[2] and Sebastián Muñoz-Guerra[1]

[1]*Departament d'Enginyeria Química, Universitat Politècnica de Catalunya, ETSEIB, Diagonal 647, 08028 Barcelona, Spain*

[2]*Departamento de Ingeniería Química, Universidad Politécnica de Madrid, ETSIIM, Gutiérrez Abascal 2, 28006 Madrid, Spain*

Corresponding author: sebastian.munoz@upc.edu

Contents:

Scheme SI-1. Synthesis of 2,4:3,5-di-*O*-methylene-D-glucitol (a), 2,4:3,5-di-*O*-methylene-D-mannitol (b).

Text: Signal assignments of the ^{1}H and ^{13}C NMR spectra recorded from polyesters.

Figure SI-1. ^{1}H NMR (top), ^{13}C (bottom) spectra of PGluxSeb homopolyester.

Figure SI-2. ^{1}H NMR (top), ^{13}C (bottom) spectra of PManxAdi homopolyester.

Figure SI-3. ^{1}H NMR (top), ^{13}C (bottom) spectra of PManxSeb homopolyester.

Figure SI-4. ^{1}H NMR (top), ^{13}C (bottom) spectra of PIsAdi homopolyester.

Figure SI-5. ^{1}H NMR (top), ^{13}C (bottom) spectra of PIsSub homopolyester.

Figure SI-6. ^{13}C-NMR spectra showing the different products generated by the transesterification of *exo* and *endo* groups of Glux-diol with dimethyl adipate.

Figure SI-7. ^{13}C-NMR spectra showing the different products generated by the transesterification of *exo* and *endo* groups of Is with dimethyl adipate.

Figure SI-8. First heating DSC traces of PGluxSeb annealed at 105 ˚C for 1 h, PGluxSub annealed at 120˚C for 1 h.

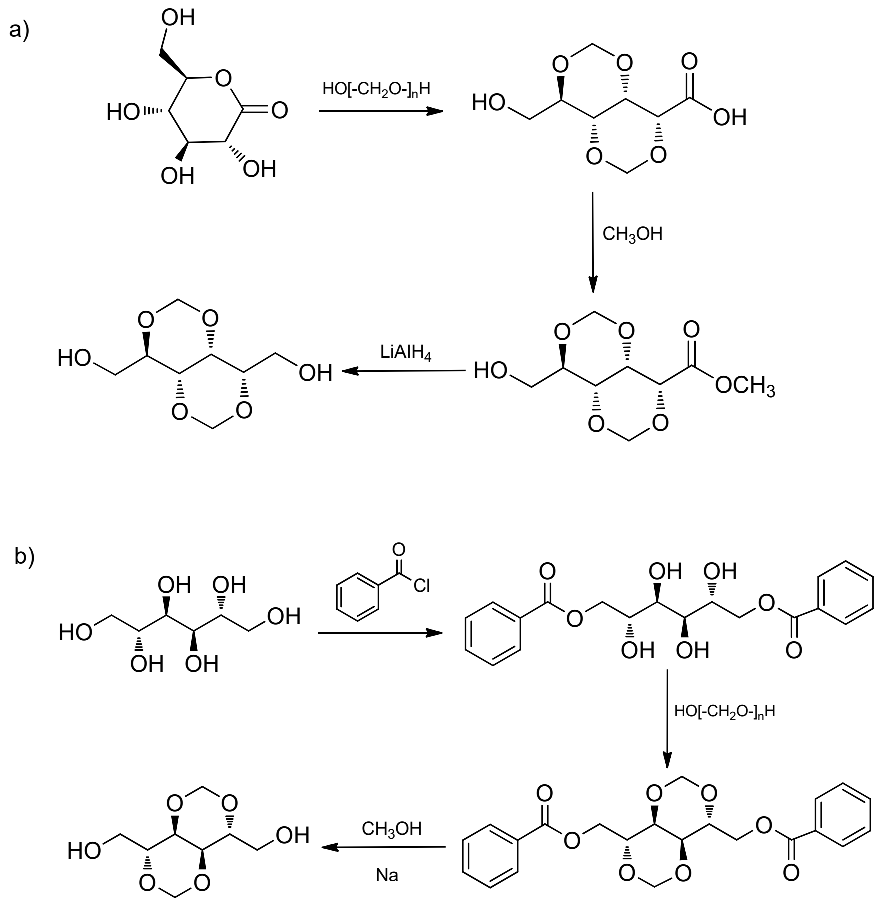

Scheme SI-1. Synthesis of 2,4:3,5-di-*O*-methylene-D-glucitol (a), 2,4:3,5-di-*O*-methylene-D-mannitol (b).

Signal assignments of the ^{1}H and ^{13}C NMR spectra recorded from polyesters.

PGluxAdi: ^{1}H NMR (300.1 MHz, $CDCl_3$), *δ* (ppm): 5.2-4.8 (m, 4H, OCH_2O), 4.6-4.2 (m, 2H, $OC\mathbf{H_2}CH$), 4.4-4.2 (m, 4H, $OC\mathbf{H_2}CH$), 4.2 (m, 1H, $OCH_2C\mathbf{H}CH$), 3.9 (m, 1H, $OCH_2C\mathbf{H}CH$), 3.8 (m, 1H, $OCH_2CHC\mathbf{H}$), 3.6 (m, 1H, $OCH_2CHCHC\mathbf{H}CH$), 2.4 (m, 4H, $COC\mathbf{H}_2CH_2$), 1.7 (m, 4H, $COCH_2C\mathbf{H_2}$). ^{13}C NMR (75.5 MHz, $CDCl_3$), *δ* (ppm): 173.0-172.8 (CO), 92.9, 88.3, 75.5, 73.8, 70.9, 67.3, 63.2, 60.5, 33.6, 24.2.

PGluxSub: ^{1}H NMR (300.1 MHz, $CDCl_3$), *δ* (ppm): 5.2-4.8 (m, 4H, OCH_2O), 4.6-4.2 (m, 2H, $OC\mathbf{H_2}CH$), 4.4-4.2 (m, 4H, $OC\mathbf{H_2}CH$), 4.2 (m, 1H, $OCH_2C\mathbf{H}CH$), 3.9 (m, 1H, $OCH_2C\mathbf{H}CH$), 3.7 (m, 1H, $OCH_2CHC\mathbf{H}$), 3.6 (m, 1H, $OCH_2CHCHC\mathbf{H}CH$), 2.3 (t, 4H, $COC\mathbf{H}_2CH_2$), 1.6 (m, 4H, $COCH_2C\mathbf{H}_2$), 1.4 (m, 4H, $COCH_2CH_2C\mathbf{H}_2$). ^{13}C NMR (75.5 MHz,

$CDCl_3$), δ (ppm): 173.5-173.2 (CO), 92.9, 88.3, 75.6, 73.8, 70.9, 67.3, 63.2, 60.3, 33.9, 28.6, 24.6.

PGluxSeb: ^{1}H NMR (300.1 MHz, $CDCl_3$), δ (ppm): 5.2-4.8 (m, 4H, OCH_2O), 4.6-4.2 (m, 2H, $OC\mathbf{H_2}CH$), 4.4-4.2 (m, 4H, $OC\mathbf{H_2}CH$), 4.2 (m, 1H, $OCH_2C\mathbf{H}CH$), 3.9 (m, 1H, $OCH_2C\mathbf{H}CH$), 3.7 (m, 1H, $OCH_2CHC\mathbf{H}$), 3.6 (m, 1H, $OCH_2CHCHC\mathbf{H}CH$), 2.3 (m, 4H, $COC\mathbf{H_2}CH_2$), 1.6 (m, 4H, $COCH_2C\mathbf{H_2}$), 1.3 (m, 8H, $COCH_2CH_2C\mathbf{H_2}C\mathbf{H_2}$). ^{13}C NMR (75.5 MHz, $CDCl_3$), δ (ppm): 173.6-173.4 (CO), 92.9, 88.3, 75.6, 73.8, 71.0, 67.3, 63.4, 34.1, 29.1, 24.8.

PManxAdi: ^{1}H NMR (300.1 MHz, $CDCl_3$), δ (ppm): 4.9-4.8 (m, 4H, OCH_2O), 4.6-4.2 (m, 4H, $OC\mathbf{H_2}CH$), 4.4 (m, 2H, $OC\mathbf{H_2}C\mathbf{H}$), 4.0 (m, 2H, $OCH_2CHC\mathbf{H}$), 2.4 (m, 4H, $COC\mathbf{H_2}CH_2$), 1.7 (m, 4H, $COCH_2C\mathbf{H_2}$). ^{13}C NMR (75.5 MHz, $CDCl_3$), δ (ppm): 173.0 (CO), 88.3, 70.8, 66.3, 63.1, 33.6, 24.2.

PManxSub: ^{1}H NMR (300.1 MHz, $CDCl_3$), δ (ppm): 4.9-4.8 (m, 4H, OCH_2O), 4.5-4.2 (m, 4H, $OC\mathbf{H_2}CH$), 4.4 (m, 2H, $OCH_2C\mathbf{H}$), 4.1 (m, 2H, $OCH_2CHC\mathbf{H}$), 2.4 (t, 4H, $COC\mathbf{H_2}CH_2$), 1.8 (m, 4H, $COCH_2C\mathbf{H_2}$), 1.3 (m, 4H, $COCH_2CH_2C\mathbf{H_2}$). ^{13}C NMR (75.5 MHz, $CDCl_3$), δ (ppm): 173.4 (CO), 88.3, 70.9, 66.4, 63.0, 34.0, 29.0, 24.6.

PManxSeb: ^{1}H NMR (300.1 MHz, $CDCl_3$), δ (ppm): 4.9-4.8 (m, 4H, OCH_2O), 4.5-4.2 (m, 4H, $OC\mathbf{H_2}CH$), 4.4 (m, 2H, $OC\mathbf{H_2}C\mathbf{H}$), 4.0 (m, 2H, $OCH_2CHC\mathbf{H}$), 2.4-2.2 (t, 4H, $COC\mathbf{H_2}CH_2$), 1.6 (m, 4H, $COCH_2C\mathbf{H_2}$), 1.3 (m, 8H, $COCH_2CH_2C\mathbf{H_2}C\mathbf{H_2}$). ^{13}C NMR (75.5 MHz, $CDCl_3$), δ (ppm): 173.6 (CO), 88.3, 70.9, 66.4, 63.0, 34.0, 29.0, 24.8.

PIsAdi: ^{1}H NMR (300.1 MHz, $CDCl_3$), δ (ppm): 5.2 (m, 1H, CHO_{exo}), 5.1 (m, 1H, CHO_{endo}), 4.8 (m, 1H, $C\mathbf{H}CHO_{endo}$), 4.6 (m, 1H, $C\mathbf{H}CHO_{exo}$), 4.0-3.7 (m, 4H, $C\mathbf{H_2}CHO_{exo}$ and $C\mathbf{H_2}CHO_{endo}$), 2.4-2.2 (m, 4H, $COC\mathbf{H_2}CH_2$), 1.7 (m, 4H, $COCH_2C\mathbf{H_2}$).^{13}C NMR (75.5 MHz, $CDCl_3$), δ (ppm): 172.6-172.2 (CO), 85.9, 80.7, 78.3, 73.9, 73.4, 70.4, 33.7-33.4, 24.2.

PIsSub: ^{1}H NMR (300.1 MHz, $CDCl_3$), δ (ppm): 5.2 (m, 1H, CHO_{exo}), 5.1 (m, 1H, CHO_{endo}), 4.8 (m, 1H, $C\mathbf{H}CHO_{endo}$), 4.5 (m, 1H, $C\mathbf{H}CHO_{exo}$), 4.0-3.7 (m, 4H, $C\mathbf{H_2}CHO_{exo}$ and $C\mathbf{H_2}CHO_{endo}$), 2.4-2.2 (m, 4H, $COC\mathbf{H_2}CH_2$), 1.6 (m, 4H, $COCH_2C\mathbf{H_2}$), 1.4 (m, 4H, $COCH_2CH_2C\mathbf{H_2}$). ^{13}C NMR (75.5 MHz, $CDCl_3$), δ (ppm): 173.0-172.6 (CO), 86.0, 80.7, 77.9, 73.8, 73.5, 70.4, 34.0-33.8, 28.7, 24.6.

PIsSeb: ^{1}H NMR (300.1 MHz, $CDCl_3$), δ (ppm): ^{1}H NMR (300.1 MHz, $CDCl_3$), δ (ppm): 5.2 (m, 1H, CHO_{exo}), 5.1 (m, 1H, CHO_{endo}), 4.8 (m, 1H, $C\mathbf{H}CHO_{endo}$), 4.5 (m, 1H, $C\mathbf{H}CHO_{exo}$), 4.0-3.7 (m, 4H, $C\mathbf{H_2}CHO_{exo}$ and $C\mathbf{H_2}CHO_{endo}$), 2.4-2.2 (m, 4H, $COC\mathbf{H_2}CH_2$), 1.6 (m, 4H, $COCH_2C\mathbf{H_2}$), 1.3 (s, 8H, $COCH_2CH_2C\mathbf{H_2}C\mathbf{H_2}$). ^{13}C NMR (75.5 MHz, $CDCl_3$), δ (ppm): 173.1-172.8 (CO), 86.0, 80.7, 77.9, 73.7, 73.5, 70.4, 34.1-33.9, 29.0, 24.8.

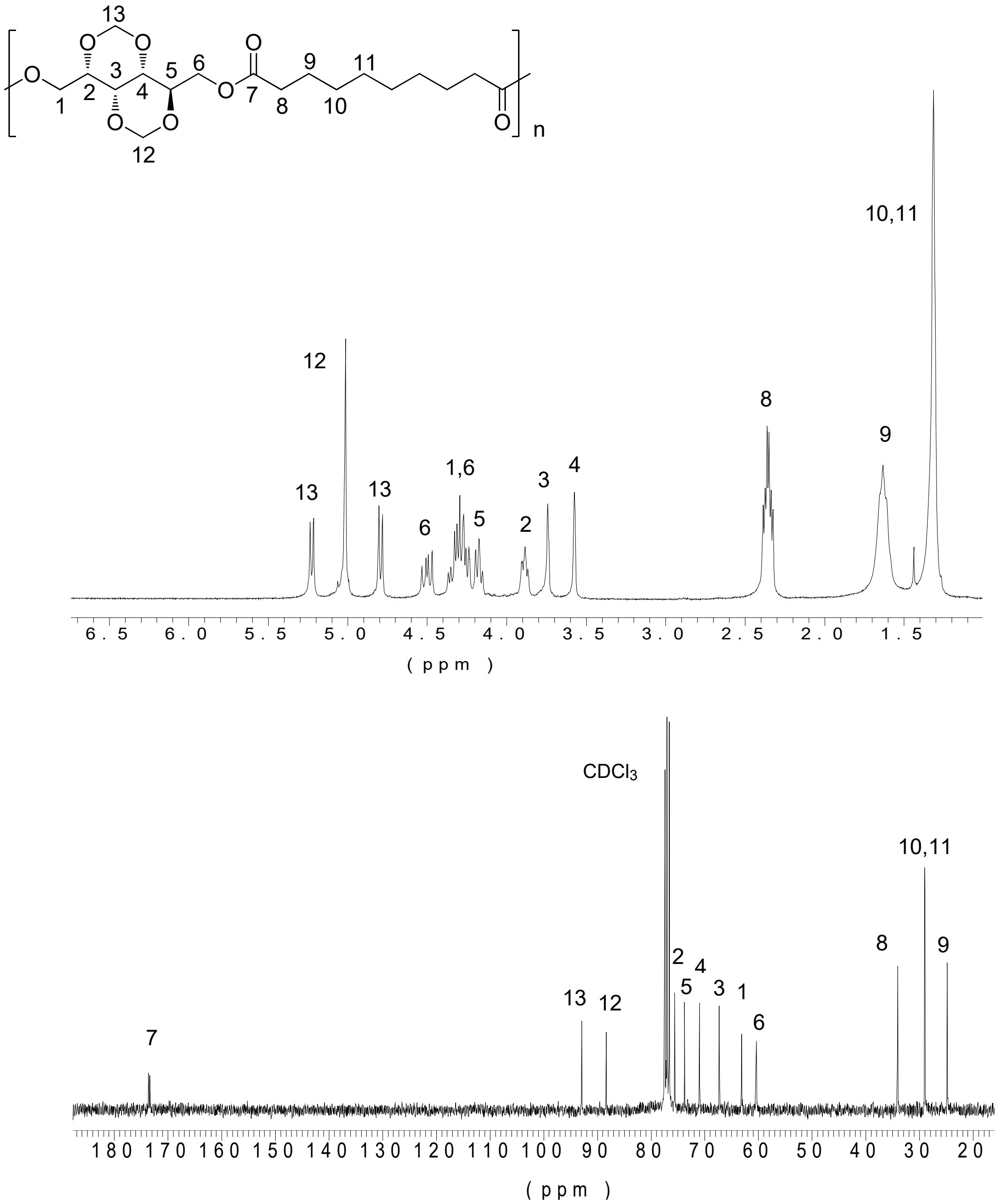

Figure SI-1. ^{1}H NMR (top), ^{13}C (bottom) spectra of PGluxSeb homopolyester.

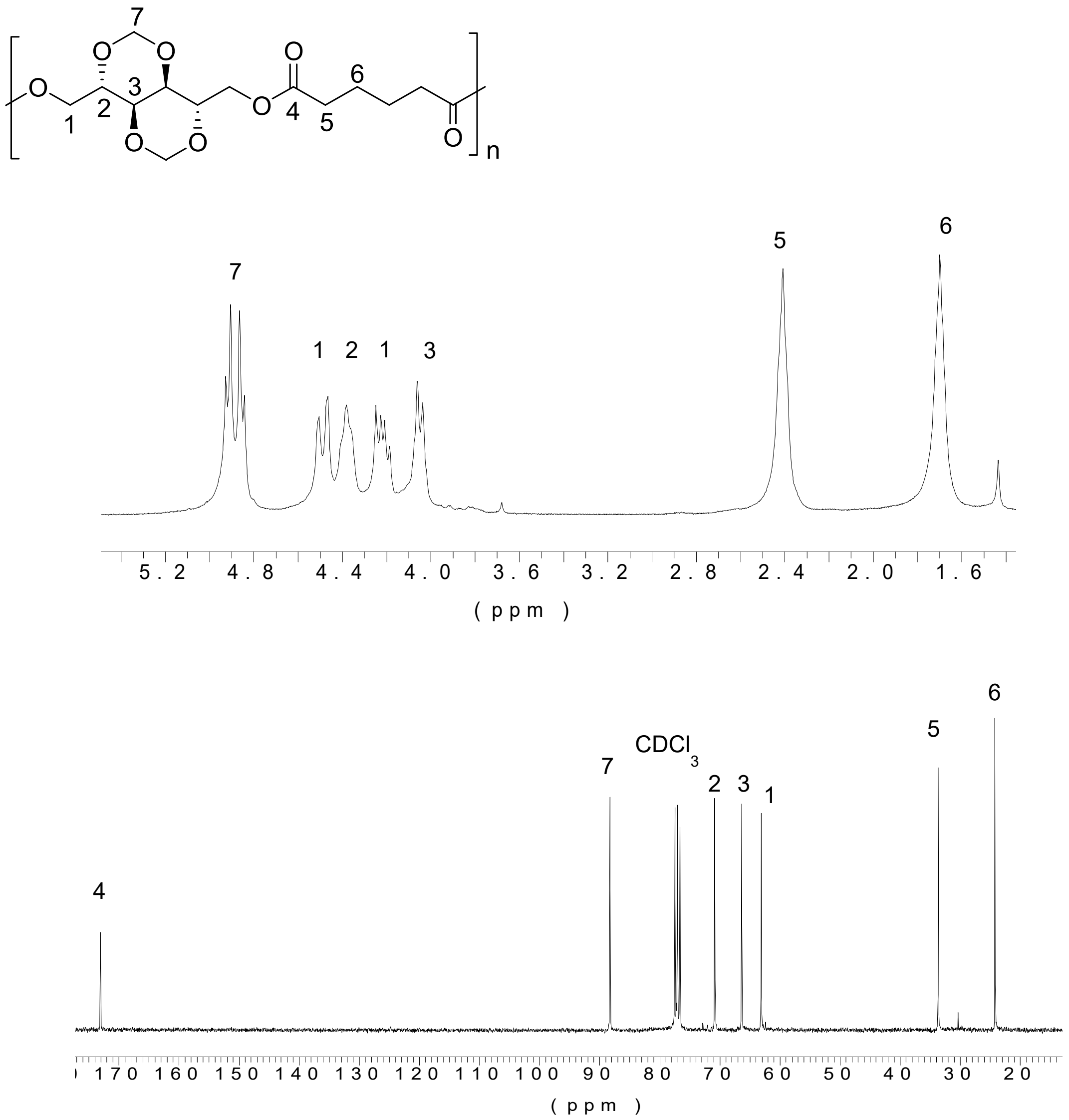

Figure SI-2. ^{1}H NMR (top), ^{13}C (bottom) spectra of PManxAdi homopolyester.

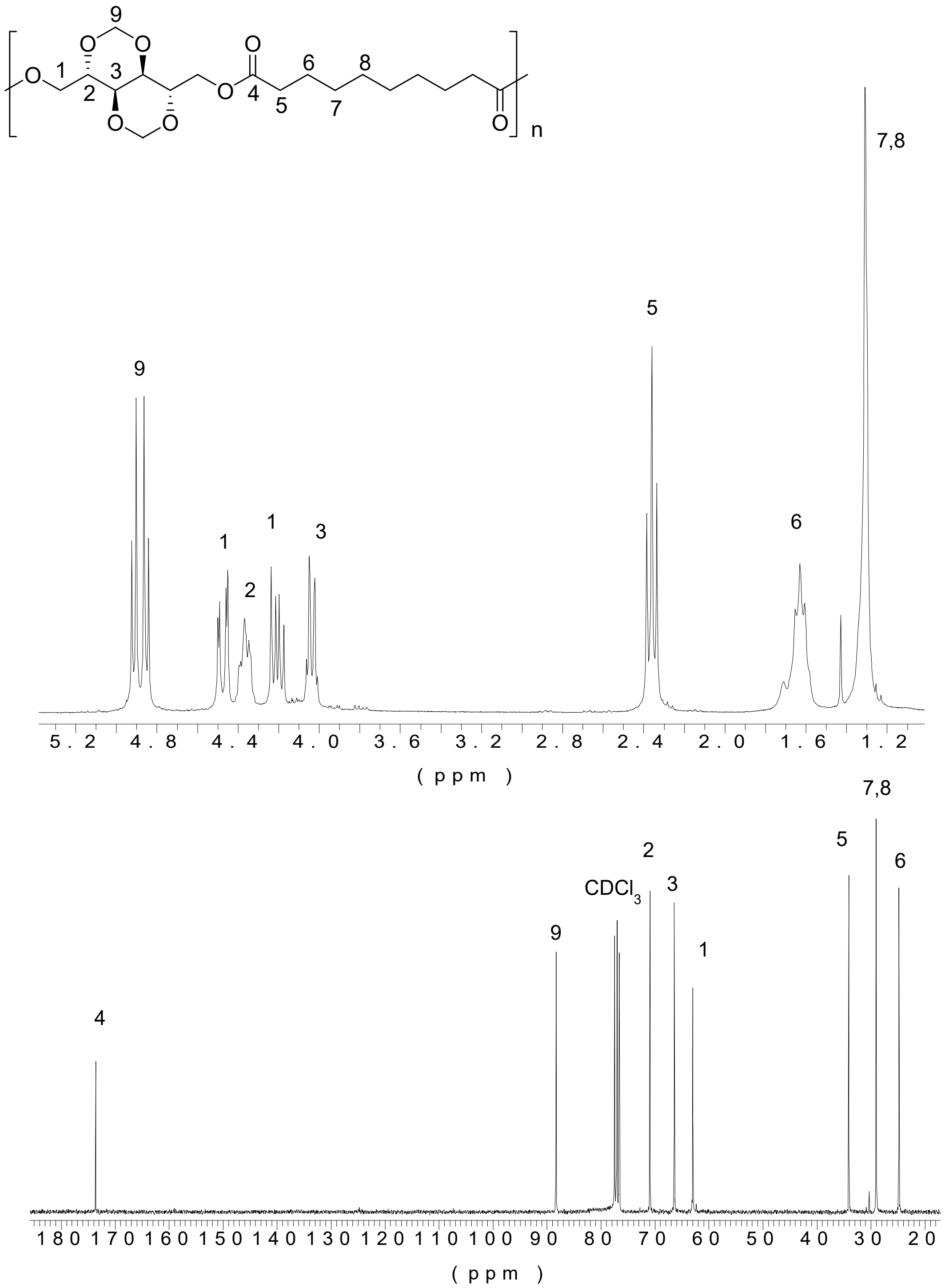

Figure SI-3. ^{1}H NMR (top), ^{13}C (bottom) spectra of PManxSeb homopolyester.

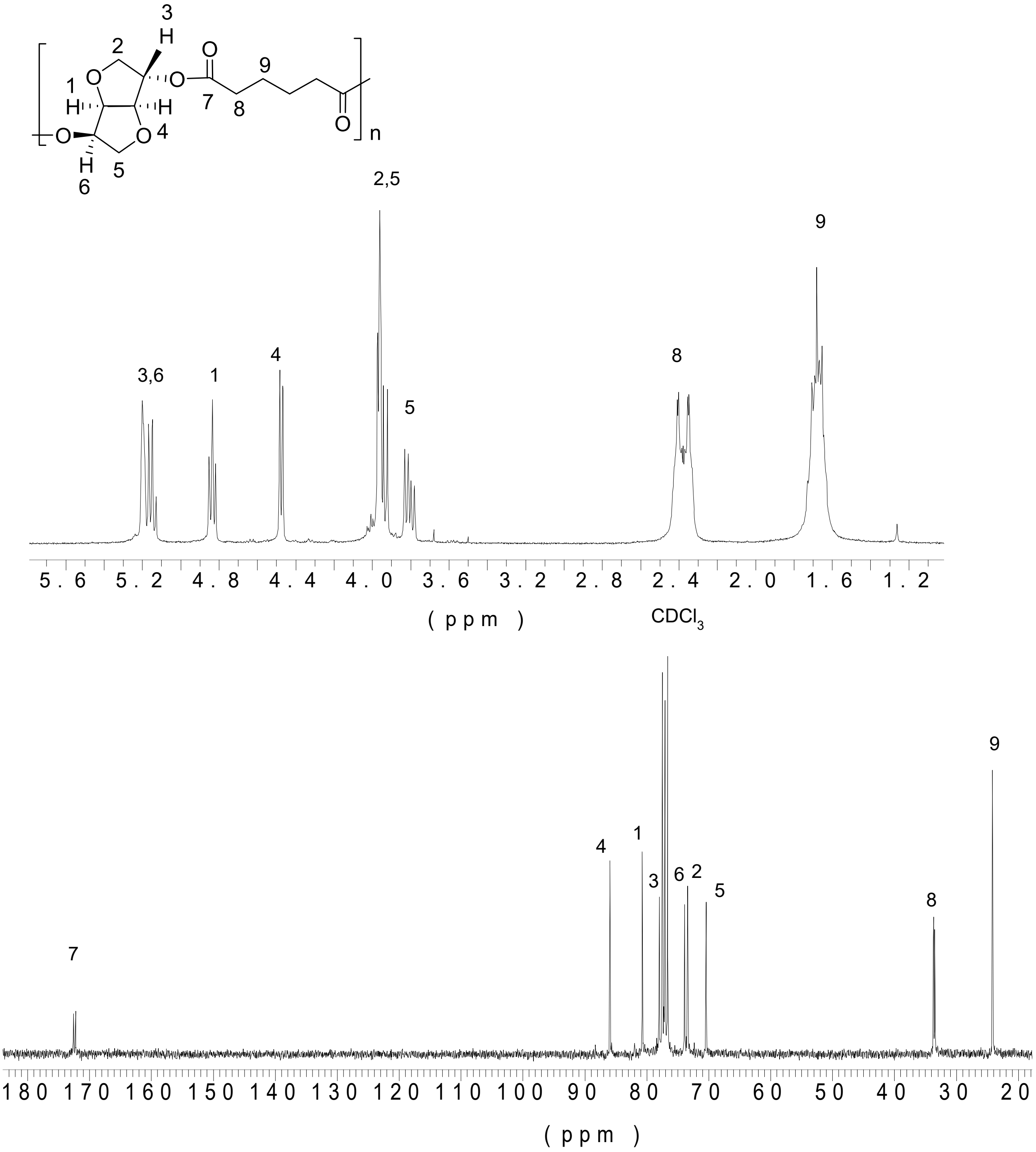

Figure SI-4. ^{1}H NMR (top), ^{13}C (bottom) spectra of PIsAdi homopolyester.

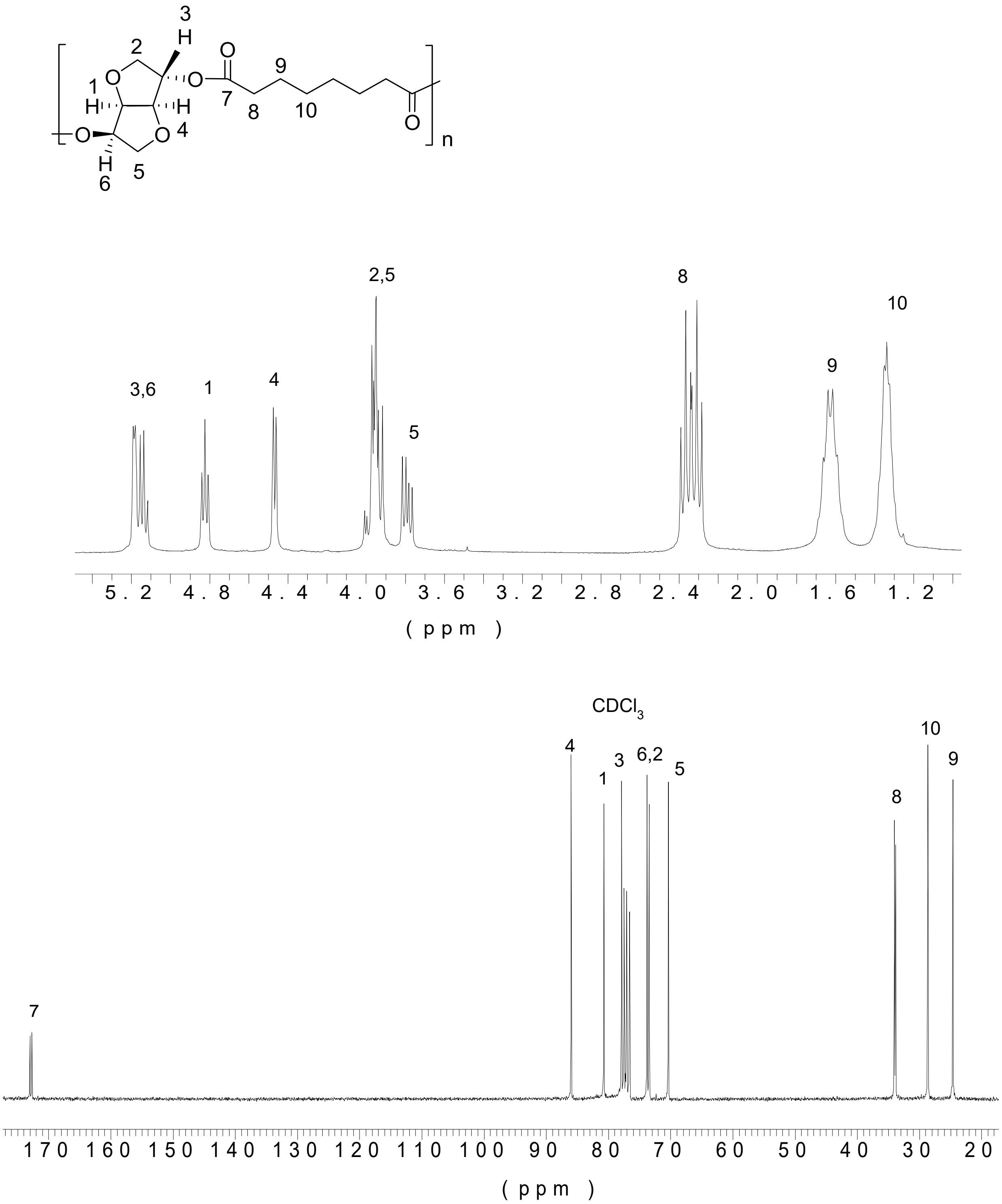

Figure SI-5. ^{1}H NMR (top) and ^{13}C (bottom) spectra of PIsSub homopolyester.

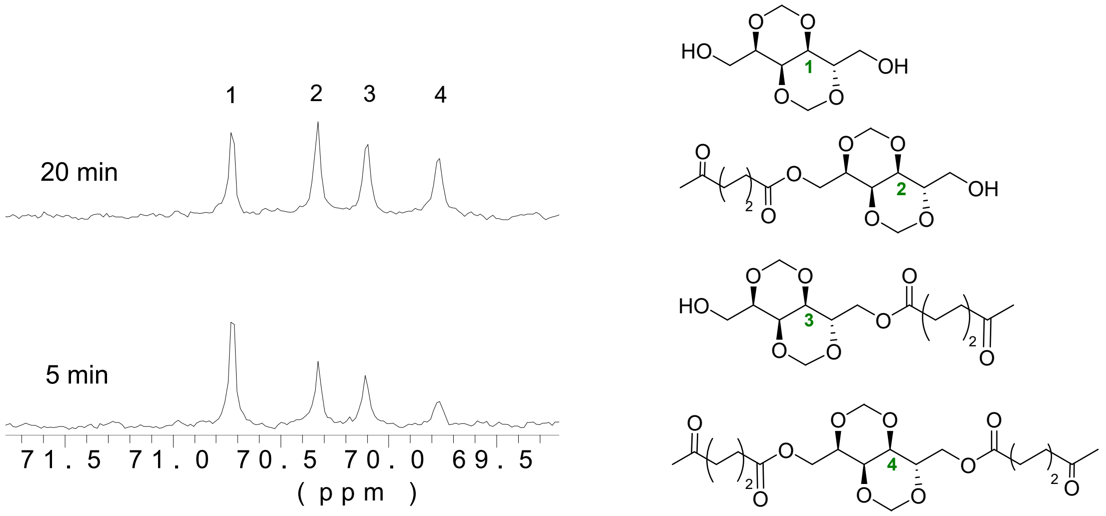

Figure SI-6. ^{13}C-NMR spectra showing the different products generated by the transesterification of *exo* and *endo* groups of Glux-diol with dimethyl adipate.

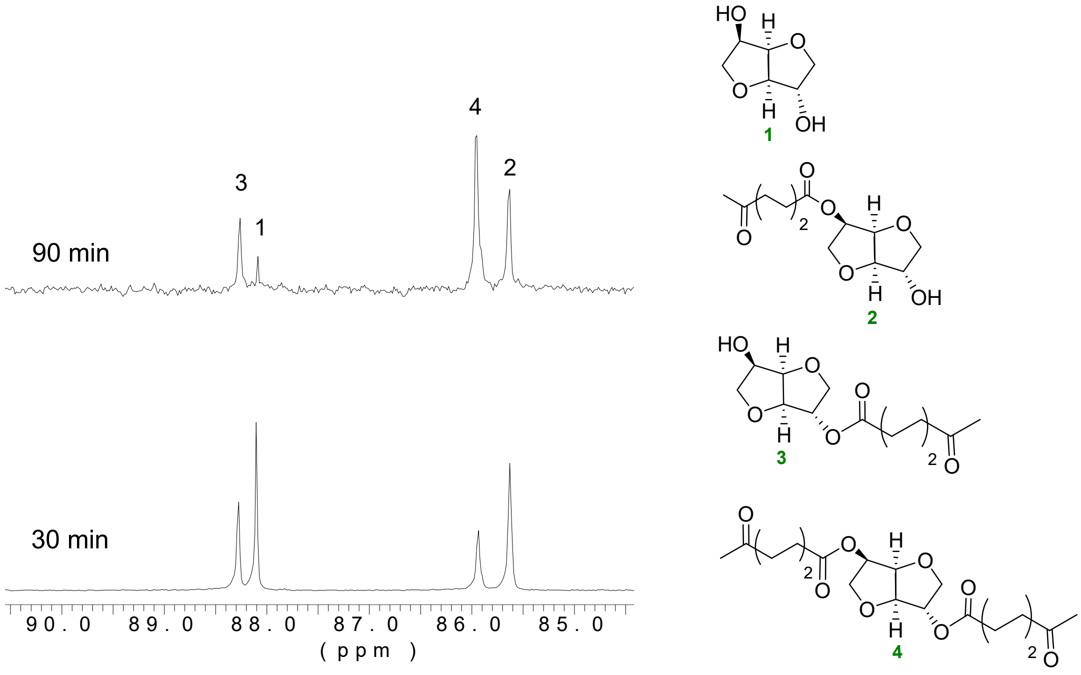

Figure SI-7. ^{13}C-NMR spectra showing the different products generated by the transesterification of *exo* and *endo* groups of Is with dimethyl adipate.

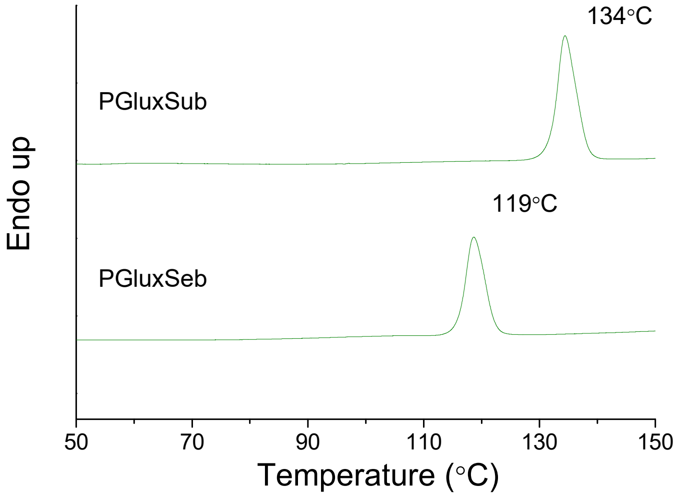

Figure SI-8. First heating DSC traces of PGluxSeb annealed at 105 ˚C for 1 h, PGluxSub annealed at 120˚C for 1 h.
